# Trace Water Changes Metal Ion Speciation in Deep Eutectic Solvents: Ce^3+^ Solvation and Nanoscale Water Clustering in Choline Chloride–Urea–Water Mixtures

**DOI:** 10.1021/acs.inorgchem.3c02205

**Published:** 2023-10-20

**Authors:** Oliver S. Hammond, Elly K. Bathke, Daniel T. Bowron, Karen J. Edler

**Affiliations:** † Centre for Sustainable Chemical Technologies, University of Bath, Claverton Down, Bath BA2 7AY, U.K.; ‡ Department of Chemistry, University of Bath, Claverton Down, Bath BA2 7AY, U.K.; § ISIS Neutron and Muon Source, Science and Technology Facilities Council, Rutherford Appleton Laboratory, Didcot OX11 0QX, U.K.

## Abstract

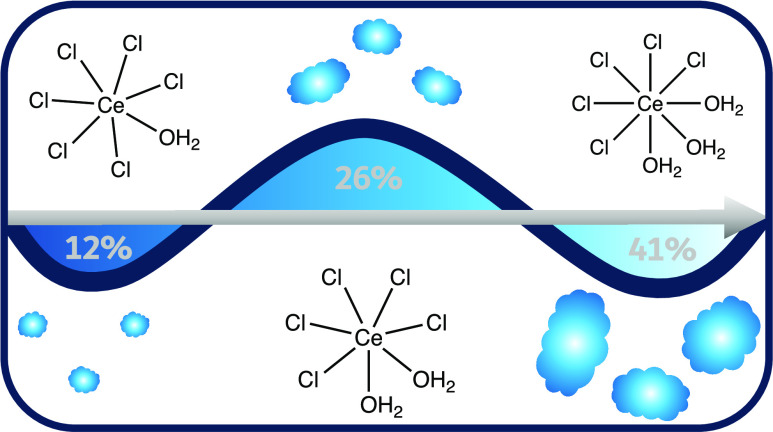

Eutectic mixtures of choline chloride, urea, and water in deep eutectic solvent (DES)/water molar hydration ratios (*w*) of 2, 5, and 10, with dissolved cerium salt, were measured using neutron diffraction with isotopic substitution. Structures were modeled using empirical potential structure refinement (EPSR). Ce^3+^ was found to form highly charged complexes with a mean coordination number between 7 and 8, with the shell containing mostly chloride, followed by water. The shell composition is strongly affected by the molar ratio of dilution, as opposed to the mass or volume fraction, due to the high affinity of Cl^–^ and H_2_O ligands that displace less favorable interactions with ligands such as urea and choline. The presence of Ce^3+^ salt disrupted the bulk DES structure slightly, making it more electrolyte-like. The measured coordination shell of choline showed significant discrepancies from the statistical noninteracting distribution, highlighting the nonideality of the blend. Cluster analysis revealed the trace presence of percolating water clusters (25 ≥ *n* ≥ 2) in solvent compositions of 5 and 10*w* for the first time.

## Abstract 2

Eutectic mixtures of choline chloride, urea, and water in DES/water molar hydration ratios (*w*) of 2, 5, and 10, with dissolved cerium salt, were measured using neutron diffraction with isotopic substitution.

## Introduction

Deep eutectic solvents (DESs) are partially ionic mixtures composed at their eutectic composition,^1^ regarded as potentially more sustainable alternative “designer solvents”.^2^ DESs are most often prepared from organic salts, such as choline chloride, and destabilizing neutral compounds with a propensity for forming a large ensemble of H-bonds, such as urea.^3−5^ A low-melting liquid state is therefore accessible for a wider catalogue of compounds than that provided by the already large array offered by pure ionic liquids (ILs) or molecular liquids (MLs).^6^ There is an accordingly wide scope for the design of a novel DES to match the requirements of almost any chemical application; DESs are becoming recognized as viable alternative solvents in the synthesis of organics,^7^ porous carbons,^8,9^ and polymers;^10,11^ in the preparation of solid-state materials such as nanoparticles,^12^ for example, via the solvothermal method;^13−17^ and in extraction,^18−20^ self-assembly of amphiphiles^21^ and polymers,^22^ protein solubilization,^23^ and electrodeposition.^24,25^

The breadth of the potential applications of DES has been widened by the recent discovery that, like ILs, multicomponent mixtures with a cosolvent (most notably DES–water mixtures) retain some characteristics of the pure system, up to a certain level.^26^ Conventional IL–cosolvent mixtures display a transition from a Coulombic-dominated medium, through a “mesophase” where molecular and ionic domains coexist in equilibrium.^27^ There is evidence for this intermediate phase in DESs, where the diluent does not significantly disrupt the dominant interactions present in the pure fluid.^28−30^ In this regime, the structure in the mixture is “plasticized” by the presence of a low-molecular-weight cosolvent, which enables strong directional structuring in the bulk^21,31−33^ or at interfaces.^34−36^ Along with the potential to use this effect to tune solvation interactions,^30,37^ this allows for tailoring the solvent environment to meet the requirements of niche applications and environmental legislation and improving basic physical properties such as viscosity^38,39^ while crucially remaining below the transition point at which the mixture becomes a simple aqueous “electrolyte solution” of DES components and its useful properties are lost.^40−42^ Research into the structure of a pure DES remains in relative infancy, with the real fundamental understanding of structure and phase behavior only starting to develop over a decade since the inception of the field.^32,33,43^ The literature regarding the behavior and structure within DES–cosolvent mixtures, specifically DES–water mixtures, is accordingly even more scarce.^21,28,29,37,44^ Therefore, there is a necessity to develop a further understanding of these systems, particularly because of the unpredictable and often nonlinear (or nonideal) trends in properties that have been observed to date.^39,41,45−48^

Ce^3+^ ions in solution are commonly applied as precursors for the production of cerium oxide nanomaterials through solvothermal and sol–gel methods, which are most famously used in catalysis applications such as automobile emission control.^49^ An interesting trend was observed for the solvothermal synthesis of nanoparticulate cerium oxide in DES, where an increase in water content increased the rate of growth of the nanostructures while also driving the formation of more desirable extended one-dimensional (1D) morphologies.^13^ This tandem size and morphological dependence of nanomaterials upon water fraction has been observed for other systems, such as iron oxide.^16^ These previous studies investigated the DES solvothermal reaction forming iron oxide nanoparticles in situ using a variety of advanced techniques, including static neutron diffraction data collected at 303 K (prereaction) for the pure choline chloride–urea DES with the cerium-nitrate precursor, which highlighted the formation of unexpected structures; a fluxional complex comprising the DES components was found to occur around Ce^3+^ ions, and the presence of strong urea–water interactions was also observed throughout the solution.^17^

Therefore, this article presents structural data showing the speciation of Ce^3+^ ions in “water-in-DES” solutions (ca. <50 wt %).^28^ The structures formed by Ce^3+^ were measured in a series of hydrated choline chloride:urea:water mixtures using neutron diffraction with isotopic substitution and resolved using empirical potential structure refinement (EPSR) modeling. These results will be discussed in the context of previous reports of anomalous reaction conditions (i.e., low temperatures) required to form high-activity nanostructures via DES solvothermal reactions.^50^ We will explore the hypothesis that this is due to unusual coordination complexes formed about rare earth centers in these mixtures.^13,51^ Herein, we show that direct experimental neutron scattering measurements can connect local Ce^3+^ ion speciation with bulk structure by giving insights into the impact of ion complexes on mean liquid structuring and interspecies interactions as a function of the DES water content.

## Experimental Section

### Preparation of Solutions

From vacuum-dried choline chloride (Fisher, ≥99%) and urea (Sigma-Aldrich, ≥99.5%), the pure eutectic mixture was first prepared by mixing under mild heating (60 °C) in the known eutectic molar ratio until a single homogeneous and transparent phase had formed. Subsequently, water (Elga, 18.2 MΩ deionized) was dosed into the prepared 1:2 DES mixture in known molar ratios, where water loading is defined as choline chloride/urea/*w* ratios, where *w* is equal to 2, 5, or 10 mol equiv of water. Thus, the samples described in this work are 1:2:2, 1:2:5, and 1:2:10 mixtures of choline chloride–urea–water referred to hereafter as reline-2*w*, reline-5*w*, and reline-10*w* for convenience, respectively. Upon addition of water, the samples were mixed at ambient conditions until the mixture appeared monophasic, signifying the complete combination of two mutually soluble liquids; no demixing was seen to occur, and the solution properties persisted irrespective of preparation via shaking, stirring, ultrasonics, or vortex mixing. After preparation of dry or hydrated stock liquids, solutions of metal ions were prepared by adding cerium(III) nitrate hexahydrate (Acros, ≥99.99% purity) in the desired concentration and mixing at room temperature to obtain a homogeneous solution. Ultraviolet–visible (UV–vis) spectroscopy measurements were performed for highly diluted solutions (at a constant Ce/ChCl/urea ratio of 0.0025:50:100) using a Thermo Scientific Evolution 201 spectrophotometer across the wavelength range of 200–900 nm with a bandwidth and data interval of 1 nm.

The process for the preparation of deuterated or partially deuterium-substituted mixtures for neutron diffraction measurements was identical, instead using combinations of *d*_9_–choline chloride (CK Isotopes, ≥99.8 atom % D), *d*_4_–urea (CK Isotopes, ≥99.8 atom % D), and D_2_O (Sigma-Aldrich, ≥99.9 atom % D). For each system measured using neutron diffraction, the samples were prepared and used immediately, with purity and isotopic substitution determined to be sufficiently high by comparing the measured (experimental) and theoretical total scattering cross sections.

### Neutron Diffraction Measurements and Atomistic Modeling

Wide *Q*-range neutron diffraction measurements of the prepared choline chloride–urea–water–cerium-nitrate solutions were performed using the NIMROD diffractometer (beamtime allocation RB1610312), located at TS2 of the STFC Rutherford Appleton Laboratory, Harwell, Oxford, U.K. NIMROD uses time-of-flight neutrons of wavelength 0.05 ≤ λ ≤ 11 Å, with detector banks spanning the angular range of 0.6–37.5°, yielding an effective *Q*-range of 0.01 ≤ *Q* ≤ 50 Å^–1^ or an approximate real-space length scale of 0.1–300 Å.^52^

Isotope-labeled samples were loaded into flat-plate sample cells of null-scattering TiZr alloy (0.68:0.32 Ti/Zr molar ratio). The cells were vacuum-sealed, with a 1 mm path length and 1 mm wall thickness, accommodating approximately 1.5 g of sample within the 30 × 30 mm NIMROD incident neutron beam footprint. Filled cells were mounted directly into a metal sample changer, throughout which a water/ethylene glycol mixture was recirculated using a Julabo heater/chiller unit to regulate the temperature to 303 ± 0.1 K at the sample positions.

In addition to the samples, measurements were performed on empty cells, the empty instrument, and a standard 3 mm thick sample of V for calibration and normalization of the instrument and data. Measurements were performed for a median of 2 h, with some variations in the counting time depending on the deuteration state of the sample. Processing of the raw data was accomplished using GudrunN software;^53^ corrections were made for the sample multiple scattering, the inherent background of the sample environment, and attenuation, and the data were then normalized to the known scattering of vanadium standard. A final component of the data reduction and correction procedure was the performance of an iterative inelastic scattering correction that is important for hydrogen-containing samples.^54^

Following corrections, data sets were modeled using Empirical Potential Structure Refinement modeling (EPSR), which has been described elsewhere^55^ and used in previous studies of DES.^3^ Lennard–Jones parameters and atom labels were identical to those used in previous publications on neutron diffraction of choline chloride–urea–water mixtures^28^ and for cerium-nitrate in anhydrous choline chloride–urea.^13^ Details of the simulation box compositions used for structure refinement are provided in the Supporting Information.

## Results and Discussion

### Fits and Data

**Figure 1:**
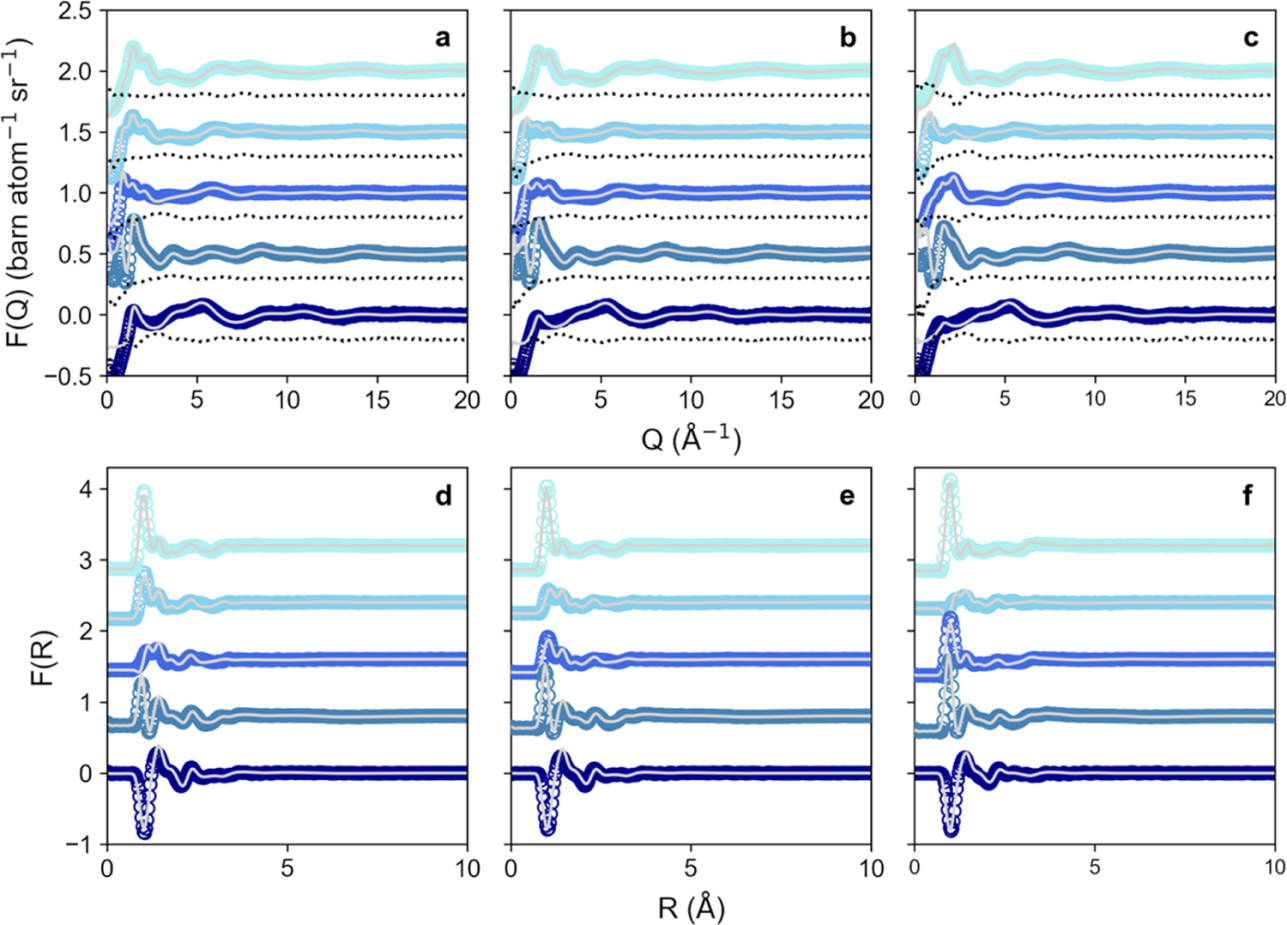
Experimental data (colored markers), fits to the data using EPSR modeling (gray lines), and fit residuals (black dotted lines) for Ce-containing ChCl/urea/water mixtures with a composition of 2*w* (a, d), 5*w* (b, e), and 10*w* (c, f), shown as a function of the scattering vector *Q* (a–c), and the same data sets and fits Fourier-transformed to real space (d–f). From top to bottom on each chart, data sets represent ChCl/urea/H_2_O neutron isotopic contrasts of D/D/D (top, light cyan), D/D/H (light blue), D/H/D (middle, royal blue), H/D/D (dark cyan), and H/H/H (bottom, dark blue).

A series of neutron diffraction experiments were performed to test our hypothesis across a range of higher water concentrations. Three water levels (2, 5, and 10*w)* and five isotopic contrasts of each (choline chloride/urea/water deuteration of H/H/H, H/D/D, D/H/D, D/D/H, and D/D/D) were measured using neutron diffraction, and the data were then modeled using EPSR. Experimental neutron diffraction data for the three different water contents are shown in Figure 1a–c, alongside the fits computed for the systems using EPSR. The quality of the fits was generally excellent, with a strong correlation between the model and data for the short-range oscillations at high values of the momentum transfer vector *Q* and only slight deviations in the low-*Q* region (*Q* ≤ 2 Å^–1^). These deviations are typical of this type of experiment and are attributable to the challenges in the treatment of the inelastic background.^54^ Real-space Fourier transforms of the fits and data are shown in Figure 1d–f.

### Ce^3+^ Local Coordination Environment

**Figure 2:**
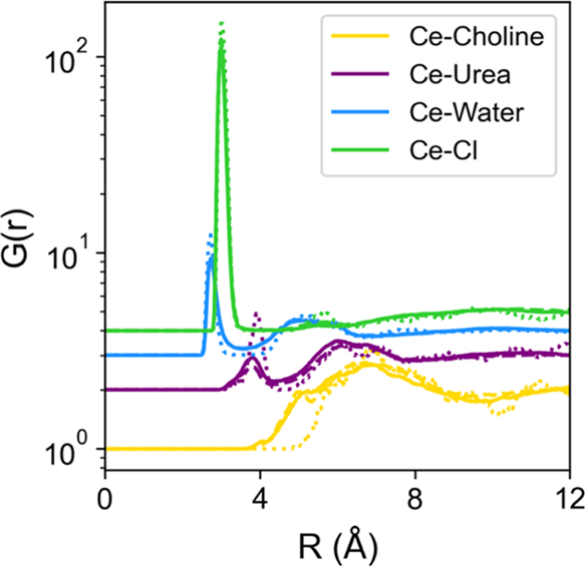
Calculated partial radial distribution functions (pRDFs) for interactions targeted on Ce^3+^ ions in DES solution. pRDFs are shown for ChCl/urea samples prepared with water contents of 2*w* (solid lines), 5*w* (dashed lines), and 10*w* (dotted lines); note: the intensity scale is logarithmic.

In the first instance, EPSR models were interrogated to obtain information about the structure of the Ce^3+^ ion in solution. In DESs, the question of coordination is quite interesting because of the rich variety of available species capable of interacting with metal centers.^17^ A set of cerium-centric partial radial distribution functions (pRDFs) was therefore calculated (Figure 2), showing the intensity and length scale of interactions with solvating species. Intermolecular coordination numbers for the various Ce^3+^ interactions were then calculated from the EPSR models by using the first pRDF minimum as the maximum radius (*R*_max_), and these are shown in Table 1. Of particular interest is that the closest molecular center of mass to Ce^3+^ ions is found to be H_2_O, with a very intense feature observed for Cl^–^ at a slightly longer range. The *N*_coord_ values for Cl^–^ and H_2_O indicate that the first solvation shell of Ce^3+^ is dominated by water and chloride. This is similar to previous observations for various different types of metal ions in DES due to their high affinity for chloride, leading to a preference toward forming the chloro complex.^17,56,57^ However, here, we observe that the relative proportion of water and Cl^–^ in the Ce^3+^ coordination shell clearly scales with the numerical composition of the components comprising the DES solution. This is with one interesting exception: for the 2*w* system, the Cl^–^ content of the shell exceeds that of pure DES, which has a slightly higher Cl^–^ concentration. This can be explained on the basis that the addition of H_2_O preferentially substitutes the bulkier, nonpreferred ligands such as urea and NO_3_^–^, which reduces the excluded volume within the ligand sphere.

**Table 1:** Average Coordination of the Cerium Ion in Various Solutions, Calculated by the Integration of Partial Radial Distribution Functions up to the First Minima Corresponding with the Primary (Inner-Sphere) Solvation Shell^a^

			*N*_coord_			
‘*A*’	‘*B*’	*R*_max_ (Å)	0*w*^b^	2*w*	5*w*	10*w*
Ce^3+^	choline	5.5	1.09 ± 0.74	0.51 ± 0.71	0.49 ± 0.64	0.22 ± 0.46
	chloride	3.5	3.90 ± 0.99	5.59 ± 0.62	5.11 ± 0.73	4.57 ± 0.58
	urea	4.3	1.67 ± 1.11	0.35 ± 0.56	0.28 ± 0.50	0.20 ± 0.42
	nitrate	4.5^b^	0.48 ± 0.50	0.00 ± 0.00	0.00 ± 0.00	0.01 ± 0.10
	water	3.5		0.93 ± 0.88	1.70 ± 1.02	2.58 ± 0.98

a Coordination numbers are calculated from the cerium ion center to the closest center-of-mass atom for each ligand species; these atom types were described as C_2N_ (choline), Cl (chloride), C_U_ (urea), N_N_ (nitrate), and O_1_ (water). Statistics were accumulated using EPSR modeling and compared with data for the pure reline-0*w* DES adapted from previously reported work.^13^ Reported errors represent one standard deviation.

b For the pure DES alone (0*w*) that was reported previously, the *R*_max_ for the Ce-nitrate coordination distance was taken as 4.2 Å.^13^

Conversely, there are minimal interactions between several of the species; for example, the long-range, broad Ce-Choline peak signifies a loose interaction between the highly charged lanthanide and the like-charged, bulky cholinium cation. An interesting observation is that the cerium-nitrate *N*_coord_ was calculated to be zero for all of these water-containing mixtures, whereas for the pure DES, this value was reported to be significantly higher at 0.48 ± 0.50, signifying a distribution between 0 and 1 bound nitrate anions in the water-free system.^13^ Similarly, the Ce–urea interaction is reduced abruptly, from a significant quantity of urea (1.67 ± 1.11) in the previously reported pure DES to merely 0.35 ± 0.56 for the 2*w* DES. Therefore, it appears that the introduction of even relatively small amounts of water, as measured by the mass or volume fraction, is quite disruptive to the formation of certain, less stable, complexes. This quantity appears to scale more closely with the water mole fraction.

**Figure 3:**
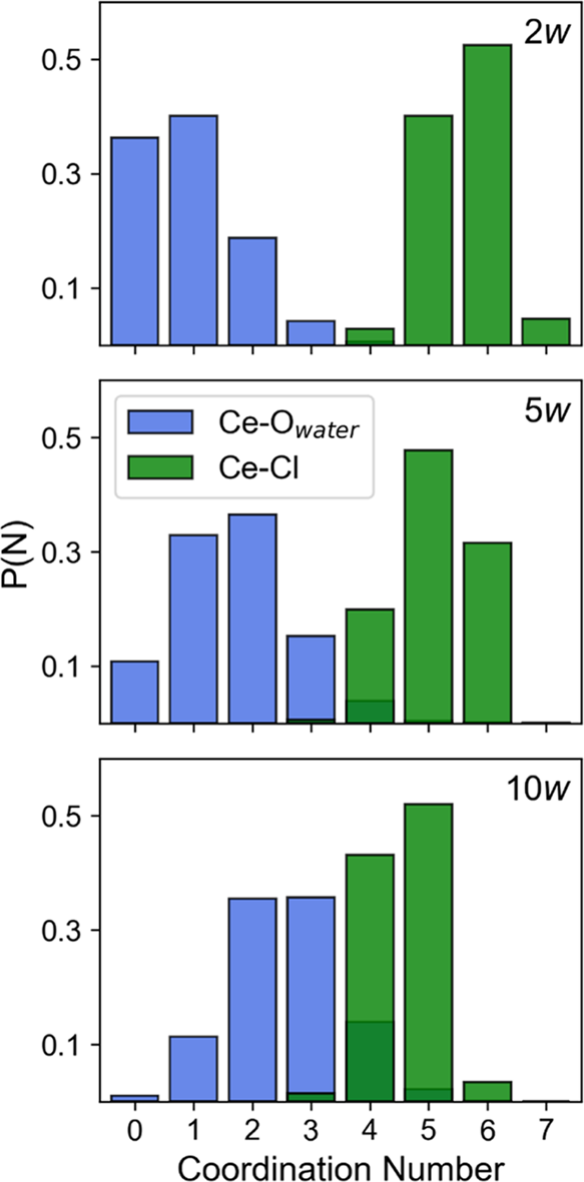
Calculated coordination number histograms showing the Ce interactions with the two most dominant ligands, H_2_O and Cl, for the 2*w* DES (top), 5*w* DES (middle), and 10*w* DES (bottom).

Coordination number histograms were therefore calculated for each species around Ce^3+^, showing that the most likely speciation in the 2*w* DES is [CeCl_6_(H_2_O)]^3–^, in the 5*w* DES is [CeCl_5_(H_2_O)_2_]^2–^, and in the 10*w* DES is [CeCl_5_(H_2_O)_3_]^2–^. The Ce–H_2_O and Ce–Cl histograms are shown in Figure 3, and those for the other species are in the Supporting Information; there is a small probability of having one or two choline or urea ligands, likely through Ce–O coordination, but never NO_3_^–^, due to the low concentration and crowding of the local environment by chloride ions and water molecules. The total coordination number of the cerium complexes thus varies between 7 and 8, i.e., the Ce^3+^ complex in the DES solution is actively in flux between geometries such as pentagonal bipyramidal, capped octahedral, or monocapped trigonal prismatic, square antiprismatic, or dodecahedral complexes, but this value is the lowest for the anhydrous DES reported previously due to the aforementioned higher excluded volume for larger species.^13^ This complexation behavior is evocative of “traditional” halometallate ionic liquids (ILs);^58^ similar structuring has been observed for various transition-metal salts in a Cl-rich DES environment^56^ and for the Ce–NO_3_ oligomeric complexes measured in halide-free “Type IV” metal-based DESs.^59^ The high ionic strength means that the coordination environment is significantly enriched in Cl^–^, with up to two-thirds of the available first neighbor sites occupied by chloride anions, and depleted in H_2_O, relative to aqueous Ln^3+^ solvation.^60,61^ The high degree of local electronic charge may make the Ce^3+^ sites more chemically active, offering an explanation for the lower temperatures observed to perform deep eutectic solvothermal synthesis.^13^ Moreover, the variation in the coordination sphere composition as a function of water content is in stark contrast to the intermolecular “DES–DES” bulk correlations, which experience hydration disruptions commensurate with the mass or volume fractions of H_2_O, rather than the mole fraction.^29^ The proposed complexation from neutron diffraction was corroborated by analysis of UV–vis spectroscopy data (Supporting Information). As the water content is systematically reduced, H_2_O is substituted by a weaker field-splitting ligand, Cl^–^, in the Ce complexes. Therefore, the energy level difference (Δ) for the coordination complexes in pure DES decreases relative to aqueous systems, which imparts a small red shift observable in the characteristic Ce^III^ absorbance bands, both at 220–230 and 300–310 nm.

**Figure 4:**
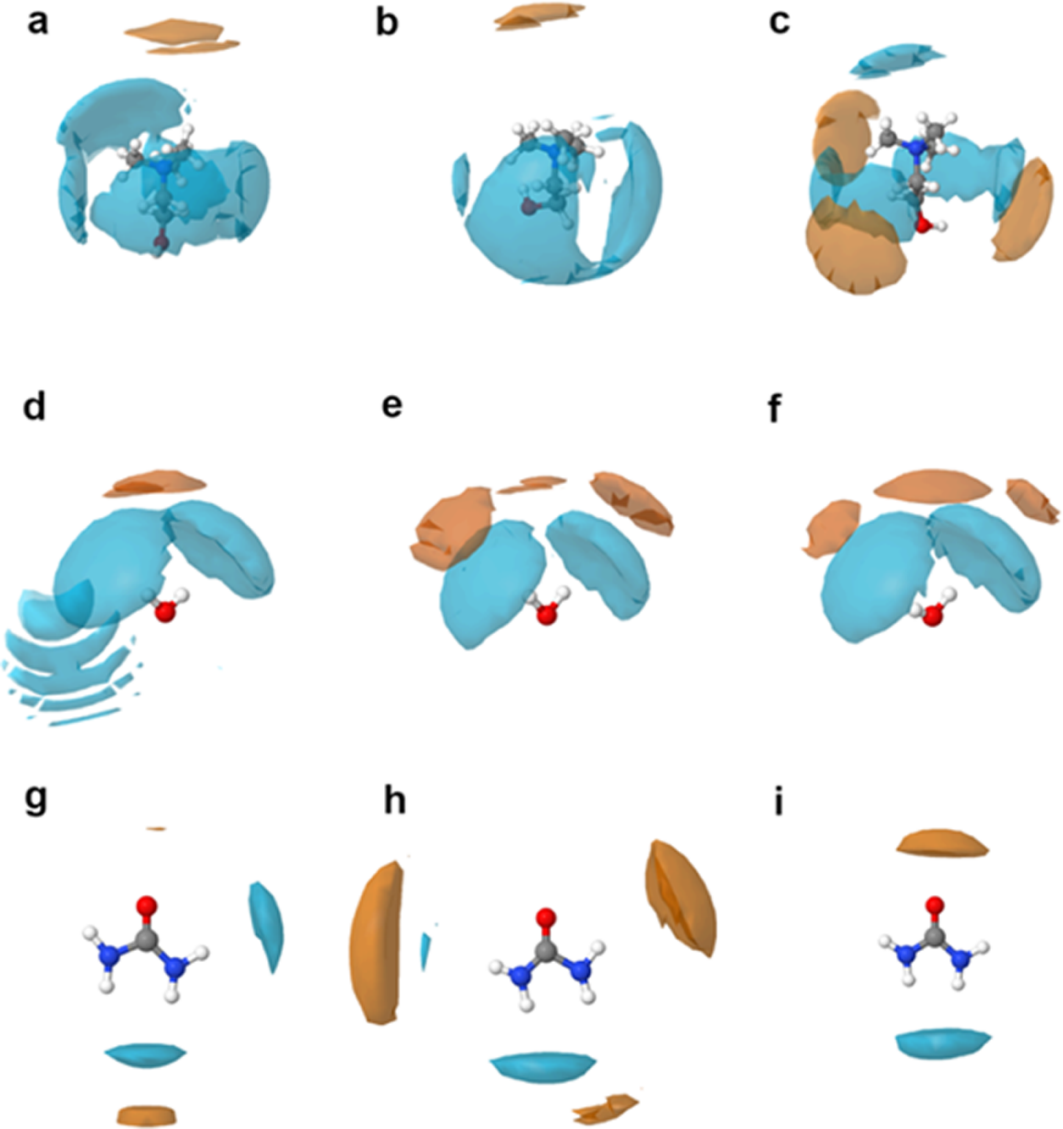
Spatial density functions (SDFs) of cerium cations (orange isosurfaces) and nitrate anions (light blue isosurfaces) in DES mixtures. Isosurfaces are plotted around choline (first row; a–c), water (second row; d–f), and urea (third row; g–i) and correspond to mixture compositions of 2*w* (first column; a–g), 5*w* (second column; b–h), and 10*w* (third column; c–i).

The preferred positions of the solutes Ce^3+^ and NO_3_^–^ around the various species in the hydrated DES solutions were determined by calculating spatial density functions (SDFs) from the EPSR simulations; these are shown in Figure 4. NO_3_^–^ is a strongly interacting H-bonding species that accepts H-bonds from the acidic protons of water, urea, and choline, as reported for structures in lanthanide-based DESs.^59^ NO_3_^–^ is thus found around these H-bonding vectors at short ranges, as well as in a broad distribution surrounding choline, which arises due to the electrostatic potential emanating from the ammonium group.^3^

### Effect of Ce^3+^ Salt on Solvent Nanostructure

The structure in the bulk was subsequently analyzed to determine the effect of adding the Ce^3+^ salt on the bulk solvent structure relative to the structuring observed in the neat DES. This is pertinent since it may significantly affect the utility and applications of DES if the structure is disrupted when they are used as solvents for reactions and processes.^37^Table 2 shows a comparison of “DES–DES” intermolecular coordination numbers (*N*_coord_) calculated with solubilized Ce^3+^ (this work) with the “metal-free” choline chloride–urea–water solutions reported in the literature.^28^ A similar, more comprehensive *N*_coord_ table for pertinent (site–site) pRDFs is given in Table S2. Broadly speaking, the addition of the metal salt appears to have a denaturing structural effect, with reference to the DES components: all interactions between DES species (i.e., choline–choline, urea–urea, choline–chloride) decrease slightly upon addition of the lanthanide salt. Meanwhile, interactions between DES components and water (i.e., chloride–water, choline–water, choline–urea, and urea–water) increase slightly in terms of coordination number, making the resulting solution slightly closer to an “aqueous” electrolyte system where the components are more strongly hydrated; the inclusion of additional ions in the solution may contribute to the overall number of formed H-bonds. This increase is, in some cases, disproportionally large, particularly for choline−water. A similar effect was observed for the structure of DES at interfaces, with an applied potential inducing more electrolyte-like structural behavior relative to the measurable layering at the open-circuit potential (OCP).^34−36^ The DES components may therefore redistribute to accommodate local maxima in charge density, in this case Ce^3+^ ions, rather than the charge state of an electrified interface, with the result being a more electrolyte-like bulk structural state. This effect was attributed to Fe^3+^ abstraction of Cl^–^ from the bulk in previous studies,^17^ but in this case, the concentration of metal salt was significantly lower.

**Table 2:** Calculated Average Intermolecular Coordination Number (*N*_coord_) Found for the Various Solutions, Calculated by the Integration of Partial Radial Distribution Functions up to the First Minima Corresponding to the Primary (Inner-Sphere) Solvation Shell^a^

						*N*_coord_		
‘*A*’	‘*B*’	*R*_max_ (Å)^b^	2*w*^c^	2*w* + Ce	5*w*^c^	5*w* + Ce	10*w*^c^	10*w* + Ce
choline	water	6.2	4.81 ± 2.22	5.03 ± 2.21	10.08 ± 3.03	10.80 ± 2.60	15.37 ± 3.08	16.67 ± 2.62
urea	water	4.9	2.62 ± 1.60	2.68 ± 1.57	5.28 ± 2.06	5.28 ± 1.85	8.09 ± 2.12	8.18 ± 1.90
chloride	water	4.5	2.17 ± 1.43	2.21 ± 1.43	4.24 ± 1.83	4.36 ± 1.62	5.78 ± 1.79	5.93 ± 1.58
water	water	4.0, 3.3, 3.1^d^	1.65 ± 1.24	1.58 ± 1.19	1.83 ± 1.08	1.61 ± 0.98	2.21 ± 1.05	2.02 ± 0.95
choline	choline	7.8	4.13 ± 1.57	3.69 ± 1.38	3.34 ± 1.56	2.80 ± 1.28	2.48 ± 1.45	2.00 ± 1.15
choline	chloride	6.4	3.01 ± 1.14	2.91 ± 1.18	2.46 ± 1.08	2.38 ± 1.13	1.87 ± 1.03	1.77 ± 1.03
choline	chloride	4.7	0.96 ± 0.77	0.92 ± 0.77	0.77 ± 0.71	0.69 ± 0.70	0.58 ± 0.65	0.49 ± 0.62
choline	urea	6.8	6.33 ± 2.12	6.86 ± 1.76	4.88 ± 1.89	5.35 ± 1.80	3.25 ± 1.63	3.43 ± 1.63
urea	chloride	5.2	1.60 ± 0.92	1.60 ± 0.93	1.26 ± 0.86	1.26 ± 0.88	0.90 ± 0.78	0.92 ± 0.79
urea	urea	5.7	3.67 ± 1.62	3.18 ± 1.38	3.09 ± 1.58	2.75 ± 1.39	2.39 ± 1.36	2.25 ± 1.33

a Coordination numbers are calculated using the closest center-of-mass atom for each ligand species; these were described as C_2N_ (choline), Cl (chloride), C_U_ (urea), N_N_ (nitrate), and O_1_ (water). Statistics were accumulated using EPSR modeling from ∼20,000 iterations of the simulation box and compared with data for cerium-free aqueous DESs from previously reported work.^28^ Reported errors represent one standard deviation.

b *R*_max_ values generally vary across a concentration series. These aqueous examples have been kept internally consistent where possible but may differ slightly from those previously reported for the 0*w* + Ce system.^13^

c Data shown are those reported previously by Hammond et al. for pure ChCl:urea:water via neutron diffraction, without solutes. *R*_max_ values are consistent with the previous reports.^28^

d The presented water–water *R*_max_ values have significant systemic variance, so they are listed.

### Nonideality in Choline Chloride–Urea–Water

**Figure 5:**
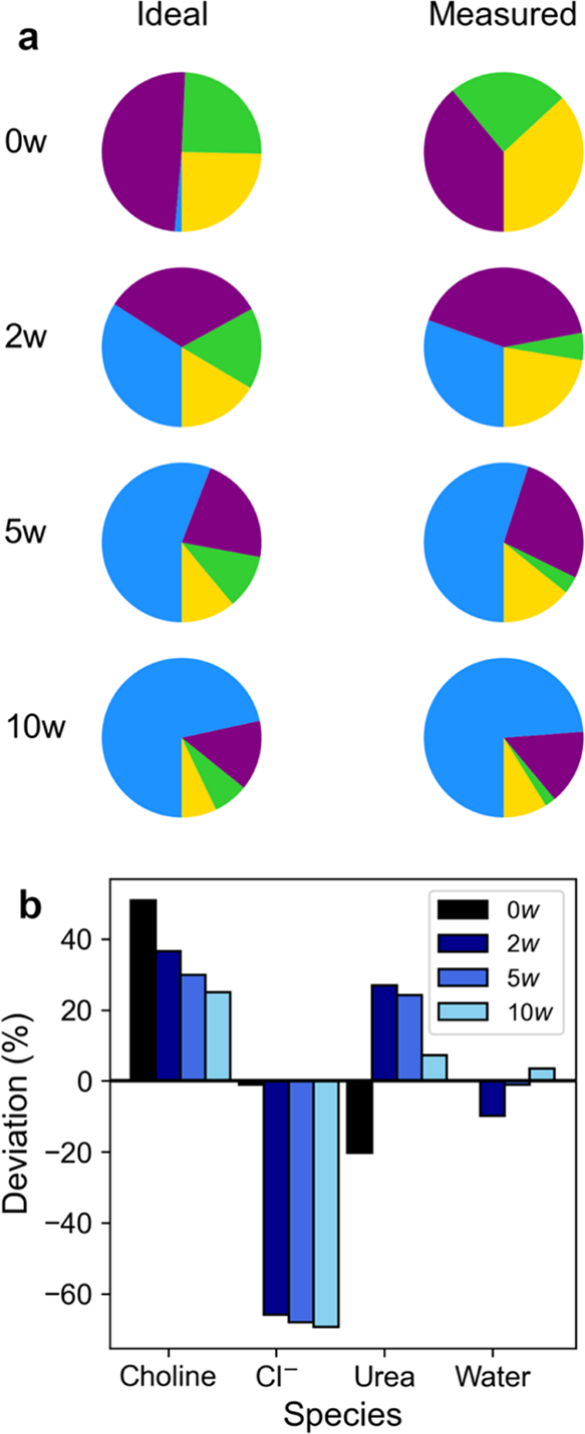
Comparison between the ideal statistical distribution of molecules around a central choline cation and those measured and fitted using neutron diffraction and EPSR. The composition of the first coordination shell (fractional coordination, i.e., ) is shown for the ideal mixture and the measured solvents (a), where blue represents water, purple represents urea, green represents chloride, and yellow represents choline. The percentage deviation in these coordination numbers is shown in (b) for the measured solvents relative to the ideal statistical distribution. For comparison, values for the 0*w* DES are plotted using values from Hammond et al.^13^ Note that the water of crystallization for the Ce salt was included in this calculation.

It has been established that DESs typically exhibit some degree of deviation from ideal mixing, with enthalpic and entropic contributions from a disordered network of strong directional H-bonds,^5^ with many available degrees of freedom,^4^ resulting in the famous excess melting point depression.^62−64^ To partially quantify the nature of this nonideality, we calculated the fractional coordination for each species around a central cholinium cation from the averages in our EPSR model and compared this to the ideal numerical distribution derived from the statistical distribution of the species used to construct the simulation box. Plots showing the fraction of each species in the first coordination sphere are shown in Figure 5a, and the percentage deviation of the experimental value from the ideal theoretical one is given in Figure 5b, respectively, for the three measured water contents, as well as a comparison with the previously reported 0*w* mixtures.^13^

The composition of choline’s first solvation shell, as determined experimentally, clearly differs from an ideal, noninteracting statistical distribution of species, as has previously been highlighted for choline chloride–malic acid–water mixtures.^30^ For these hydrated samples, this shell appears to be enriched in other choline cations and urea molecules but very strongly depleted in terms of Cl^–^. This provides support for a structural picture where Cl^–^ is pulled away from its cation, frustrating crystallization in the mixture. The degree of chloride depletion increases with the solvent water content, while the enrichment of choline and urea decreases. At 2*w*, the solvation shell of choline is slightly depleted of water, but this flips to becoming slightly enriched at 10*w*. Clearly, these mixtures are composed of a series of strongly interacting and mutually miscible components,^65^ which do not mix in an ideal manner.

### Water Clustering in Choline Chloride–Urea–Water

The idea that nanoscale clusters of water exist in the DES has been an area of debate for some time. Some evidence, typically from spectroscopic measurements such as PFG-NMR, has been provided in support of this, showing, for example, differing diffusion coefficients for the different components; this has variably been interpreted as water “channels”, or even as a spongelike (*L*_3_-type) subphase of water.^25,47,66−68^ Small, transient nanoscale H_2_O clusters such as this are well known in mixtures of water with various hydrophilic and hydrophobic molecular species.^69−73^ However, no significant small-angle signal has been observed in previous measurements of water in DESs using wide *Q*-range neutron diffraction with isotopic substitution of typical hydrophilic DES systems based on choline chloride salts.^3,17,28−30^ Clusters have previously been reported, but this was for amphiphilic DESs, tailored to observe percolation and pseudophase segregation of long alkyl domains.^74^

**Figure 6:**
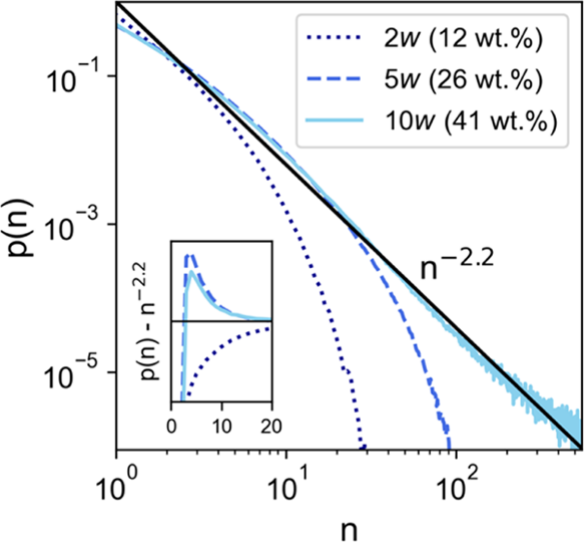
Cluster size probability distributions *p*(*n*) calculated for water molecules in the bulk DES mixtures of cluster size n for DES compositions of 2*w* (navy dotted line), 5*w* (royal blue dashed line), and 10*w* (light blue solid line). Cluster probabilities were calculated from EPSR simulations using the cluster formation distance threshold of 1 Å ≤ O_1_···H_1_ ≤ 2.5 Å. A power law line (black, solid) following the probability decay of *n*^–2.2^ is plotted alongside,^75^ representing the random 3D bulk percolation threshold. Inset: Cluster sizes (abscissa; linear scale) following subtraction of the power law percolation threshold line to emphasize observation of small clusters, with a tie line drawn on the ordinate at *p*(*n*) = 0.

Cluster size probability distributions for H_2_O in the three DES mixtures were therefore calculated and are shown in Figure 6. A percolating cluster is observed when the probability distribution of that cluster breaches the random 3D percolation threshold, *N*α*S*^–2.2^.^75^ The inset also shows the probability distributions after subtraction of the percolation threshold line to emphasize the crossover point. The 2*w* system never shows any percolation. However, 5 and 10*w* DESs both exceed the percolation threshold line by a small fraction at around 25 ≥ *n* ≥ 2, where *n* is the number of H_2_O molecules in the percolating cluster. Therefore, this is the first direct structural experimental evidence of transient water cluster formation in a hydrophilic DES, but it must be highlighted that the fraction of percolating clusters is very small such that it cannot be directly observed in the diffraction signal, likely falling short of being a true *L*_3_ phase. The cluster distribution probably comprises several different favorable configurations. For example, there may be a small quantity of transient “channels”, although these are likely to be in constant flux,^47,66−68^ and they may also take the form of “pearl-on-string” assemblies with the participation of Cl^–^, as has been demonstrated computationally and experimentally.^41,76^ This reinforces the view that local compositional (solvation) effects are achievable even in highly hydrophilic DES systems.^30,37,65^

## Conclusions

We therefore present evidence that Ce^3+^ forms anionic coordination complexes in DESs where the coordination sphere contains mostly Cl^–^ and H_2_O, scaling with the mole fraction of water; for a 2*w* DES, the complex formed is [CeCl_6_(H_2_O)]^3–^, for 5*w*, it is [CeCl_5_(H_2_O)_2_]^2–^, and at 10*w* of hydration, [CeCl_5_(H_2_O)_3_]^2^ is found. The results presented here are of significant interest to broader rare earth (RE) research due to parallels with the structural richness of lanthanide complexes in (solid and liquid phase) halide-saturated systems^77,78^ and their relevance for developing advanced extraction processes,^79^ such as LiCl anion exchange used in lanthanide/actinide isotope production.^80^

One of the aims of this study was to test the hypothesis that supramolecular preorganization of atypical metal ligands, such as urea coordination complexes, can reduce the activation barrier for solvothermal reactions in DES.^13^ The Ce^3+^ complexes we observed in DES have a high charge density, which may explain the heightened solvothermal reactivity. Very small mass fractions of added water were found to disrupt the complexation seen in pure DESs because the presence of trace moisture provides a molar excess of high-affinity ligands, which readily displace more esoteric ones.

Therefore, DESs remain exciting as media for coordination and extraction chemistry, but we highlight that trace quantities of water can significantly alter the solvation sphere, which could alter performance and results, and thus this must be tightly controlled in any applications involving both DES and the presence of metal ions. This finding is a major contrast to previous observations that the DES bulk structure varies in accordance with the volume fraction (i.e., proportional to the mass fraction), not the mole fraction, of added water.^28,29^

Investigation of the bulk solvent structure reveals subtle differences upon addition of the rare earth salt: chloride has a very high affinity for Ce^3+^, so it is abstracted away from the bulk. Simultaneously, the introduction of highly charge-dense metal centers appears to drive the DES components to associate slightly more strongly with water, making the resulting solutions more “aqueous-like”, in the same way that applying a potential to an electrochemical interface has been observed to interrupt DES ordering.^34^

Deeper interrogation of the EPSR models yielded evidence for negative deviations from ideal mixing but also crucially for the formation of a small proportion of transient but percolating nanoscale water clusters, which have dimensions of between 2 and 25 water molecules for the 5 and 10*w*, but not 2*w*, eutectic mixtures. These have been predicted by various techniques but, so far, have not been observed by purely structure-sensitive techniques (i.e., diffraction) in such hydrophilic DES systems. This can be explained by the transient nature of such clusters, with only a small fraction percolating at any given time. The findings presented here are therefore important for the further development of methodologies using DES; trace water strongly alters specific metal ion-DES structuring, which, depending on the application, could be crucial or pernicious.

## Supporting Information Available

Dimensions of EPSR simulation boxes, full set of partial radial distribution functions (pRDFs) and associated coordination numbers (*N*_coord_); coordination histograms of urea or chloride around Ce^3+^, and UV–vis spectroscopy data (PDF)

## References

[ref1] Kollau L. J. B. M.; Vis M.; van den Bruinhorst A.; Esteves A. C. C.; Tuinier R. Quantification of the Liquid Window of Deep Eutectic Solvents. Chem. Commun. 2018, 54 (95), 13351–13354. 10.1039/C8CC05815F. 30417900

[ref2] Hansen B. B.; Spittle S.; Chen B.; Poe D.; Zhang Y.; Klein J. M.; Horton A.; Adhikari L.; Zelovich T.; Doherty B. W.; Gurkan B.; Maginn E. J.; Ragauskas A.; Dadmun M.; Zawodzinski T. A.; Baker G. A.; Tuckerman M. E.; Savinell R. F.; Sangoro J. R. Deep Eutectic Solvents: A Review of Fundamentals and Applications. Chem. Rev. 2021, 121 (3), 1232–1285. 10.1021/acs.chemrev.0c00385. 33315380

[ref3] Hammond O. S.; Bowron D. T.; Edler K. J. Liquid Structure of the Choline Chloride-Urea Deep Eutectic Solvent (Reline) from Neutron Diffraction and Atomistic Modelling. Green Chem. 2016, 18, 2736–2744. 10.1039/C5GC02914G.

[ref4] Ashworth C. R.; Matthews R. P.; Welton T.; Hunt P. A. Doubly Ionic Hydrogen Bond Interactions within the Choline Chloride–Urea Deep Eutectic Solvent. Phys. Chem. Chem. Phys. 2016, 18, 18145–18160. 10.1039/C6CP02815B. 27328990

[ref5] Araujo C. F.; Coutinho J. A. P.; Nolasco M. M.; Parker S. F.; Ribeiro-Claro P. J. A.; Rudić S.; Soares B. I. G.; Vaz P. D. Inelastic Neutron Scattering Study of Reline: Shedding Light on the Hydrogen Bonding Network of Deep Eutectic Solvents. Phys. Chem. Chem. Phys. 2017, 19 (27), 17998–18009. 10.1039/C7CP01286A. 28665431

[ref6] Angell C. A.; Ansari Y.; Zhao Z.; et al. Ionic Liquids: Past, Present and Future. Faraday Discuss. 2012, 154, 9–27. 10.1039/C1FD00112D. 22455011

[ref7] Alonso D. A.; Baeza A.; Chinchilla R.; Guillena G.; Pastor I. M.; Ramón D. J. Deep Eutectic Solvents: The Organic Reaction Medium of the Century. Eur. J. Org. Chem. 2016, 2016 (4), 612–632. 10.1002/ejoc.201501197.

[ref8] López-Salas N.; Carriazo D.; Gutiérrez M. C.; Ferrer M. L.; Ania C. O.; Rubio F.; Tamayo A.; Fierro J. L. G.; del Monte F. Tailoring the Textural Properties of Hierarchical Porous Carbons Using Deep Eutectic Solvents. J. Mater. Chem. A 2016, 4 (23), 9146–9159. 10.1039/C6TA02704K.

[ref9] Kapilov-Buchman K.; Portal L.; Zhang Y.; Fechler N.; Antonietti M.; Silverstein M. S. Hierarchically Porous Carbons from an Emulsion-Templated, Urea-Based Deep Eutectic. J. Mater. Chem. A 2017, 5 (31), 16376–16385. 10.1039/C7TA01958K.

[ref10] Mota-Morales J. D.; Sánchez-Leija R. J.; Carranza A.; Pojman J. A.; del Monte F.; Luna-Bárcenas G. Free-Radical Polymerizations of and in Deep Eutectic Solvents: Green Synthesis of Functional Materials. Prog. Polym. Sci. 2018, 78, 139–153. 10.1016/j.progpolymsci.2017.09.005.

[ref11] Carriazo D.; Serrano M. C.; Gutiérrez M. C.; Ferrer M. L.; del Monte F. Deep-Eutectic Solvents Playing Multiple Roles in the Synthesis of Polymers and Related Materials. Chem. Soc. Rev. 2012, 41 (14), 4996–5014. 10.1039/c2cs15353j. 22695767

[ref12] Hammond O. S.; Mudring A.-V. Ionic Liquids and Deep Eutectics as a Transformative Platform for the Synthesis of Nanomaterials. Chem. Commun. 2022, 58, 3865–3892. 10.1039/D1CC06543B. 35080210

[ref13] Hammond O. S.; Edler K. J.; Bowron D. T.; Torrente-Murciano L. Deep Eutectic-Solvothermal Synthesis of Nanostructured Ceria. Nat. Commun. 2017, 8, 14150 10.1038/ncomms14150. 28120829 PMC5288492

[ref14] Datta S.; Jo C.; De Volder M.; Torrente-Murciano L. Morphological Control of Nanostructured V _2_ O _5_ by Deep Eutectic Solvents. ACS Appl. Mater. Interfaces 2020, 12 (16), 18803–18812. 10.1021/acsami.9b17916. 32212670

[ref15] Exposito A. J.; Barrie P. J.; Torrente-Murciano L. Fast Synthesis of CeO _2_ Nanoparticles in a Continuous Microreactor Using Deep Eutectic Reline As Solvent. ACS Sustainable Chem. Eng. 2020, 8 (49), 18297–18302. 10.1021/acssuschemeng.0c06949.

[ref16] Hammond O. S.; Eslava S.; Smith A. J.; Zhang J.; Edler K. J. Microwave-Assisted Deep Eutectic-Solvothermal Preparation of Iron Oxide Nanoparticles for Photoelectrochemical Solar Water Splitting. J. Mater. Chem. A 2017, 5, 16189–16199. 10.1039/C7TA02078C.

[ref17] Hammond O. S.; Atri R. S.; Bowron D. T.; de Campo L.; et al. Structural Evolution of Iron Forming Iron Oxide in a Deep Eutectic-Solvothermal Reaction. Nanoscale 2021, 13, 1723–1737. 10.1039/D0NR08372K. 33428701

[ref18] Li C.; Zhang J.; Li Z.; Yin J.; Cui Y.; Liu Y.; Yang G. Extraction Desulfurization of Fuels with ‘Metal Ions’ Based Deep Eutectic Solvents (MDESs). Green Chem. 2016, 18 (13), 3789–3795. 10.1039/C6GC00366D.

[ref19] Damilano G.; Laitinen A.; Willberg-Keyriläinen P.; Lavonen T.; Häkkinen R.; Dehaen W.; Binnemans K.; Kuutti L. Effects of Thiol Substitution in Deep-Eutectic Solvents (DESs) as Solvents for Metal Oxides. RSC Adv. 2020, 10 (39), 23484–23490. 10.1039/D0RA03696J. 35520324 PMC9054895

[ref20] Peeters N.; Binnemans K.; Riaño S. Solvometallurgical Recovery of Cobalt from Lithium-Ion Battery Cathode Materials Using Deep-Eutectic Solvents. Green Chem. 2020, 22 (13), 4210–4221. 10.1039/D0GC00940G. PMC9426644 36132435

[ref21] Manasi I.; Bryant S. J.; Hammond O. S.; Edler K. J. Interactions of Water and Amphiphiles with Deep Eutectic Solvent Nanostructures. In Eutectic Solvents and Stress in Plants; Elsevier, 2021; p 28.

[ref22] Sapir L.; Stanley C. B.; Harries D. Properties of Polyvinylpyrrolidone in a Deep Eutectic Solvent. J. Phys. Chem. A 2016, 120 (19), 3253–3259. 10.1021/acs.jpca.5b11927. 26963367

[ref23] Sanchez-Fernandez A.; Edler K. J.; Arnold T.; Alba Venero D.; Jackson A. J. Protein Conformation in Pure and Hydrated Deep Eutectic Solvents. Phys. Chem. Chem. Phys. 2017, 19, 8667–8670. 10.1039/C7CP00459A. 28300267

[ref24] Abbott A. P.; McKenzie K. J. Application of Ionic Liquids to the Electrodeposition of Metals. Phys. Chem. Chem. Phys. 2006, 8, 4265–4279. 10.1039/b607329h. 16986069

[ref25] Abbott A.; Aldous L.; Borisenko N.; Coles S.; Fontaine O.; Gamarra Garcia J. D.; Gardas R.; Hammond O.; Hardwick L. J.; Haumesser P.-H.; Hausen F.; Horwood C.; Jacquemin J.; Jones R.; Jónsson E.; Lahiri A.; MacFarlane D.; Marlair G.; May B.; Medhi H.; Paschoal V. H.; Reid J. E. S. J.; Schoetz T.; Tamura K.; Thomas M. L.; Tiwari S.; Uralcan B.; van den Bruinhorst A.; Watanabe M.; Wishart J. Electrochemistry: General Discussion. Faraday Discuss. 2018, 206, 405–426. 10.1039/C7FD90093G. 29186221

[ref26] Kohno Y.; Ohno H. Ionic Liquid/Water Mixtures: From Hostility to Conciliation. Chem. Commun. 2012, 48 (57), 7119–7130. 10.1039/c2cc31638b. 22683915

[ref27] MacFarlane D. R.; Chong A. L.; Forsyth M.; Kar M.; Ranganathan V.; Somers A.; Pringle J. M. New Dimensions in Salt-Solvent Mixtures: A 4th Evolution of Ionic Liquids. Faraday Discuss. 2018, 206, 9–28. 10.1039/C7FD00189D. 29034392

[ref28] Hammond O. S.; Bowron D. T.; Edler K. J. The Effect of Water upon Deep Eutectic Solvent Nanostructure: An Unusual Transition from Ionic Mixture to Aqueous Solution. Angew. Chem., Int. Ed. 2017, 56, 9782–9785. 10.1002/anie.201702486. PMC5596335 28480595

[ref29] Hammond O. S.; Bowron D. T.; Jackson A. J.; Arnold T.; Sanchez-Fernandez A.; Tsapatsaris N.; Sakai V. G.; Edler K. J. Resilience of Malic Acid Natural Deep Eutectic Solvent Nanostructure to Solidification and Hydration. J. Phys. Chem. B 2017, 121, 7473–7483. 10.1021/acs.jpcb.7b05454. 28699758

[ref30] Hammond O. S.; Atri R.; Bowron D. T.; Edler K. J. Neutron Diffraction Study of Indole Solvation in Deep Eutectic Systems of Choline Chloride, Malic Acid, and Water. Chem. - Eur. J. 2022, 28, e202200566 10.1002/chem.202200566. 35510678 PMC9400976

[ref31] Kaur S.; Gupta A.; Kashyap H. K. How Hydration Affects the Microscopic Structural Morphology in a Deep Eutectic Solvent. J. Phys. Chem. B 2020, 124 (11), 2230–2237. 10.1021/acs.jpcb.9b11753. 32105490

[ref32] Kaur S.; Kumari M.; Kashyap H. K. Microstructure of Deep Eutectic Solvents: Current Understanding and Challenges. J. Phys. Chem. B 2020, 124 (47), 10601–10616. 10.1021/acs.jpcb.0c07934. 33151072

[ref33] Hammond O. S.; Edler K. J. Structure and Implications. In Deep Eutectic Solvents: Synthesis, Properties, and Applications; Wiley-VCH: Weinheim, 2019; pp 25–42.

[ref34] Hammond O. S.; Li H.; Westermann C.; Endres F.; Al-Murshedi A. Y. M.; Abbott A. P.; Warr G.; Edler K. J.; Atkin R. Nanostructure of the Deep Eutectic Solvent/Platinum Electrode Interface as a Function of Potential and Water Content. Nanoscale Horiz. 2019, 4, 158–168. 10.1039/C8NH00272J. 32254151

[ref35] Elbourne A.; Meftahi N.; Greaves T. L.; McConville C. F.; Bryant G.; Bryant S. J.; Christofferson A. J. Nanostructure of a Deep Eutectic Solvent at Solid Interfaces. J. Colloid Interface Sci. 2021, 591, 38–51. 10.1016/j.jcis.2021.01.089. 33592524

[ref36] Elbourne A.; Besford Q. A.; Meftahi N.; Crawford R. J.; Daeneke T.; Greaves T. L.; McConville C. F.; Bryant G.; Bryant S. J.; Christofferson A. J. The Impact of Water on the Lateral Nanostructure of a Deep Eutectic Solvent–Solid Interface. Aust. J. Chem. 2022, 75, 111–125. 10.1071/CH21078.

[ref37] Hammond O. S.; Simon G.; Gomes M. C.; Padua A. A. H. Tuning the Solvation of Indigo in Aqueous Deep Eutectics. J. Chem. Phys. 2021, 154, 224502 10.1063/5.0051069. 34241234

[ref38] Dai Y.; Witkamp G.-J.; Verpoorte R.; Choi Y. H. Tailoring Properties of Natural Deep Eutectic Solvents with Water to Facilitate Their Applications. Food Chem. 2015, 187, 14–19. 10.1016/j.foodchem.2015.03.123. 25976992

[ref39] Meng X.; Ballerat-Busserolles K.; Husson P.; Andanson J.-M. Impact of Water on the Melting Temperature of Urea + Choline Chloride Deep Eutectic Solvent. New J. Chem. 2016, 40 (5), 4492–4499. 10.1039/C5NJ02677F.

[ref40] Hammond O. S.; Bowron D. T.; Edler K. J. Effect of Water upon Deep Eutectic Solvent Nanostructure: An Unusual Transition from Ionic Mixture to Aqueous Solution. Angew. Chem. 2017, 129, 9914–9917. 10.1002/ange.201702486. PMC5596335 28480595

[ref41] Di Pietro M. E.; Hammond O.; van den Bruinhorst A.; Mannu A.; Padua A.; Mele A.; Gomes M. C. Connecting Chloride Solvation with Hydration in Deep Eutectic Systems. Phys. Chem. Chem. Phys. 2021, 23, 107–111. 10.1039/D0CP05843B. 33346262

[ref42] López-Salas N.; Vicent-Luna J. M.; Imberti S.; Posada E.; Roldán M. J.; Anta J. A.; Balestra S. R. G.; Madero Castro R. M.; Calero S.; Jiménez-Riobóo R. J.; Gutiérrez M. C.; Ferrer M. L.; del Monte F. Looking at the “Water-in-Deep-Eutectic-Solvent” System: A Dilution Range for High Performance Eutectics. ACS Sustainable Chem. Eng. 2019, 7 (21), 17565–17573. 10.1021/acssuschemeng.9b05096.

[ref43] Abranches D. O.; Coutinho J. A. P. Type V Deep Eutectic Solvents: Design and Applications. Curr. Opin. Green Sustainable Chem. 2022, 35, 100612 10.1016/j.cogsc.2022.100612.

[ref44] Miao S.; Jiang H. J.; Imberti S.; Atkin R.; Warr G. Aqueous Choline Amino Acid Deep Eutectic Solvents. J. Chem. Phys. 2021, 154 (21), 214504 10.1063/5.0052479. 34240972

[ref45] Posada E.; López-Salas N.; Riobóo R. J. J.; Ferrer M. L.; Gutiérrez M. C.; del Monte F. Reline Aqueous Solutions Behaving as Liquid Mixtures of H-Bonded Co-Solvents: Microphase Segregation and Formation of Co-Continuous Structures as Indicated by Brillouin and ^1^ H NMR Spectroscopies. Phys. Chem. Chem. Phys. 2017, 19 (26), 17103–17110. 10.1039/C7CP02180A. 28636032

[ref46] Kollau L. J. B. M.; Vis M.; van den Bruinhorst A.; de With G.; Tuinier R. Activity Modelling of the Solid–Liquid Equilibrium of Deep Eutectic Solvents. Pure Appl. Chem. 2019, 91 (8), 1341–1349. 10.1515/pac-2018-1014. PMC11670871 39726941

[ref47] AlZahrani Y. M.; Britton M. M. Probing the Influence of Zn and Water on Solvation and Dynamics in Ethaline and Reline Deep Eutectic Solvents by ^1^ H Nuclear Magnetic Resonance. Phys. Chem. Chem. Phys. 2021, 23 (38), 21913–21922. 10.1039/D1CP03204F. 34559172

[ref48] Guajardo N.; de María H. P. D.; Ahumada K.; Schrebler R. A.; Ramírez-Tagle R.; Crespo F. A.; Carlesi C. Water as Cosolvent: Nonviscous Deep Eutectic Solvents for Efficient Lipase-Catalyzed Esterifications. ChemCatChem 2017, 9 (8), 1393–1396. 10.1002/cctc.201601575.

[ref49] Kašpar J.; Fornasiero P.; Graziani M. Use of CeO2-Based Oxides in the Three-Way Catalysis. Catal. Today 1999, 50 (2), 285–298. 10.1016/S0920-5861(98)00510-0.

[ref50] Siegel A. L.; Adhikari L.; Salik S.; Baker G. A. Progress and Prospects for Deep Eutectic Solvents in Colloidal Nanoparticle Synthesis. Curr. Opin. Green Sustainable Chem. 2023, 41, 100770 10.1016/j.cogsc.2023.100770.

[ref51] Amphlett J. T. M.; Lee Y.; Yang W.; Kang D.; Sung N.-E.; Park J.; Jung E. C.; Choi S. Spectroscopic Study into Lanthanide Speciation in Deep Eutectic Solvents. ACS Omega 2022, 7 (1), 921–932. 10.1021/acsomega.1c05386. 35036756 PMC8756809

[ref52] Bowron D. T.; Soper A. K.; Jones K.; Ansell S.; Birch S.; Norris J.; Perrott L.; Riedel D.; Rhodes N. J.; Wakefield S. R.; Botti A.; Ricci M.-A.; Grazzi F.; Zoppi M. NIMROD: The Near and InterMediate Range Order Diffractometer of the ISIS Second Target Station. Rev. Sci. Instrum. 2010, 81 (3), 033905 10.1063/1.3331655. 20370190

[ref53] Soper A. K. GudrunN and GudrunX: Programs for Correcting Raw Neutron and X-Ray Diffraction Data to Differential Scattering Cross Section, Rutherford Appleton Laboratory Technical Report RAL-TR-–0132011, 2011.

[ref54] Soper A. K. Inelasticity Corrections for Time-of-Flight and Fixed Wavelength Neutron Diffraction Experiments. Mol. Phys. 2009, 107 (16), 1667–1684. 10.1080/00268970903025667.

[ref55] Soper A. Empirical Potential Structure Refinement. http://www.isis.stfc.ac.uk/groups/disordered-materials/downloads/empirical-potential-structure-refinement6157.html.

[ref56] Hartley J. M.; Ip C. M.; Forrest G. C. H.; Singh K.; Gurman S. J.; Ryder K. S.; Abbott A. P.; Frisch G. EXAFS Study into the Speciation of Metal Salts Dissolved in Ionic Liquids and Deep Eutectic Solvents. Inorg. Chem. 2014, 53, 6280–6288. 10.1021/ic500824r. 24897923

[ref57] Abbott A. P.; Al-Barzinjy A. A.; Abbott P. D.; Frisch G.; Harris R. C.; Hartley J.; Ryder K. S. Speciation, Physical and Electrolytic Properties of Eutectic Mixtures Based on CrCl3·6H2O and Urea. Phys. Chem. Chem. Phys. 2014, 16 (19), 9047–9055. 10.1039/c4cp00057a. 24695874

[ref58] Estager J.; Holbrey J. D.; Swadźba-Kwaśny M. Halometallate Ionic Liquids – Revisited. Chem. Soc. Rev. 2014, 43 (3), 847–886. 10.1039/C3CS60310E. 24189615

[ref59] Hammond O. S.; Bowron D. T.; Edler K. J. Structure and Properties of “Type IV” Lanthanide Nitrate Hydrate:Urea Deep Eutectic Solvents. ACS Sustainable Chem. Eng. 2019, 7 (5), 4932–4940. 10.1021/acssuschemeng.8b05548.

[ref60] Díaz-Moreno S.; Ramos S.; Bowron D. T. Solvation Structure and Ion Complexation of La3+ in a 1 Molal Aqueous Solution of Lanthanum Chloride. J. Phys. Chem. A 2011, 115 (24), 6575–6581. 10.1021/jp202961t. 21574644

[ref61] Fernández-Ramírez E.; Jiménez-Reyes M.; Solache-Ríos M. J. Stability Constants of Chloride Complexes of Lanthanum. J. Chem. Eng. Data 2007, 52 (2), 373–376. 10.1021/je060290k.

[ref62] Kollau L. J. B. M.; Vis M.; van den Bruinhorst A.; Tuinier R.; de With G. Entropy Models for the Description of the Solid-Liquid Regime of Deep Eutectic Solutions. J. Mol. Liq. 2020, 302, 112155 10.1016/j.molliq.2019.112155.

[ref63] Martins M. A. R.; Pinho S. P.; Coutinho J. A. P. Insights into the Nature of Eutectic and Deep Eutectic Mixtures. J. Solution Chem. 2019, 48, 962–982. 10.1007/s10953-018-0793-1.

[ref64] Coutinho J. A. P.; Pinho S. P. Special Issue on Deep Eutectic Solvents: A Foreword. Fluid Phase Equilib. 2017, 448, 1 10.1016/j.fluid.2017.06.011. PMC6161808 30270965

[ref65] Zahn S. Deep Eutectic Solvents: Similia Similibus Solvuntur?. Phys. Chem. Chem. Phys. 2017, 19, 4041–4047. 10.1039/C6CP08017K. 28111663

[ref66] D’Agostino C.; Gladden L. F.; Mantle M. D.; Abbott A. P.; Ahmed; Essa I.; Al-Murshedi A. Y. M.; Harris R. C. Molecular and Ionic Diffusion in Aqueous – Deep Eutectic Solvent Mixtures: Probing Inter-Molecular Interactions Using PFG NMR. Phys. Chem. Chem. Phys. 2015, 17, 15297–15304. 10.1039/C5CP01493J. 25994171

[ref67] Häkkinen R.; Alshammari O.; Timmermann V.; D’Agostino C.; Abbott A. Nanoscale Clustering of Alcoholic Solutes in Deep Eutectic Solvents Studied by Nuclear Magnetic Resonance and Dynamic Light Scattering. ACS Sustainable Chem. Eng. 2019, 7 (17), 15086–15092. 10.1021/acssuschemeng.9b03771.

[ref68] Abbott A. P.; Alabdullah S. S. M.; Al-Murshedi A. Y. M.; Ryder K. S. Brønsted Acidity in Deep Eutectic Solvents and Ionic Liquids. Faraday Discuss. 2018, 206, 365–377. 10.1039/C7FD00153C. 28926059

[ref69] Bowron D. T. Comprehensive Structural Modelling of Aqueous Solutions Using Neutron Diffraction and X-Ray Absorption Spectroscopy. J. Phys.: Conf. Ser. 2009, 190, 012022 10.1088/1742-6596/190/1/012022.

[ref70] Bowron D. T.; Finney J. L.; Soper A. K. Structural Characteristics of a 0.23 Mole Fraction Aqueous Solution of Tetrahydrofuran at 20 Degrees C. J. Phys. Chem. B 2006, 110 (41), 20235–20245. 10.1021/jp064170v. 17034201

[ref71] Towey J. J.; Soper A. K.; Dougan L. What Happens to the Structure of Water in Cryoprotectant Solutions?. Faraday Discuss. 2014, 167, 159–176. 10.1039/c3fd00084b. 24640490

[ref72] Towey J. J.; Soper A. K.; Dougan L. Molecular Insight Into the Hydrogen Bonding and Micro-Segregation of a Cryoprotectant Molecule. J. Phys. Chem. B 2012, 116 (47), 13898–13904. 10.1021/jp3093034. 23101974

[ref73] Soper A. K.; Castner E. W.; Luzar A. Impact of Urea on Water Structure: A Clue to Its Properties as a Denaturant?. Biophys. Chem. 2003, 105, 649–666. 10.1016/S0301-4622(03)00095-4. 14499925

[ref74] McDonald S.; Murphy T.; Imberti S.; Warr G. G.; Atkin R. Amphiphilically Nanostructured Deep Eutectic Solvents. J. Phys. Chem. Lett. 2018, 9, 3922–3927. 10.1021/acs.jpclett.8b01720. 29961321

[ref75] Jan N. Large Lattice Random Site Percolation. Phys. A 1999, 266, 72–75. 10.1016/S0378-4371(98)00577-9.

[ref76] Sapir L.; Harries D. Restructuring a Deep Eutectic Solvent by Water: The Nanostructure of Hydrated Choline Chloride/Urea. J. Chem. Theory Comput. 2020, 16 (5), 3335–3342. 10.1021/acs.jctc.0c00120. 32223260

[ref77] Evans W. J.; Shreeve J. L.; Ziller J. W.; Doedens R. J. Structural Diversity in Solvated Lanthanide Halide Complexes. Inorg. Chem. 1995, 34 (3), 576–585. 10.1021/ic00107a009.

[ref78] Hines C. C.; Cordes D. B.; Griffin S. T.; Watts S. I.; Cocalia V. A.; Rogers R. D. Flexible Coordination Environments of Lanthanide Complexes Grown from Chloride-Based Ionic Liquids. New J. Chem. 2008, 32 (5), 872–877. 10.1039/b800045j.

[ref79] Nockemann P.; Thijs B.; Lunstroot K.; Parac-Vogt T. N.; Görller-Walrand C.; Binnemans K.; Van Hecke K.; Van Meervelt L.; Nikitenko S.; Daniels J.; Hennig C.; Van Deun R. Speciation of Rare-Earth Metal Complexes in Ionic Liquids: A Multiple-Technique Approach. Chem. - Eur. J. 2009, 15 (6), 1449–1461. 10.1002/chem.200801418. 19123214

[ref80] Hulet E. K.; Gutmacher R. G.; Coops M. S. Group Separation of the Actinides from the Lanthanides by Anion Exchange. J. Inorg. Nucl. Chem. 1961, 17 (3–4), 350–360. 10.1016/0022-1902(61)80161-9.

