# Supplementary material for: Trace Water Changes Metal Ion Speciation in Deep Eutectic Solvents: Ce3+ Solvation and Nanoscale Water Clustering in Choline Chloride–Urea–Water Mixtures
Source: Inorg Chem. 2023 Oct 20;62(44):18069–78. doi: 10.1021/acs.inorgchem.3c02205 (PMC10630939; doi:10.1021/acs.inorgchem.3c02205)
Supplement: Supplementary file 1 — ic3c02205_si_001.pdf [file ic3c02205_si_001.pdf]

Supporting Information for:

Trace Water Changes Metal Ion Speciation in Deep Eutectic Solvents: Ce^{3+} Solvation and Nanoscale Water Clustering in Choline Chloride–Urea–Water Mixtures

Oliver S. Hammond,[1,2,4] Elly K Bathke,[1,2,5] Daniel T. Bowron,[3] and Karen J. Edler[1,2,5]*

[1]Centre for Sustainable Chemical Technologies, University of Bath, Claverton Down, Bath BA2 7AY, UK.

[2]Department of Chemistry, University of Bath, Claverton Down, Bath BA2 7AY, UK.

[3]ISIS Neutron and Muon Source, Science and Technology Facilities Council, Rutherford Appleton Laboratory, Didcot OX11 0QX, UK.

[4]Current address: Department of Biological and Chemical Engineering, Aarhus University, Aarhus C 8000, Denmark.

[5]Current address: Centre for Analysis and Synthesis, Lund University, Lund 22362, Sweden.

Email: oliver.s.hammond@bath.edu

Table S1. Chosen EPSR simulation box compositions for the three different aqueous DES systems studied here (*w* = 2, 5, 10), and number of accumulated model iterations.

w	**Ce^{3+}**	**NO_3^-**	**H_2O**	**[Cholinium]$^+$**	**Cl^-**	**Urea**	**Molecules**	**Atoms**	**Iterations**
0§	2	6	12	215	215	430	880	8226	~6000
2	5	15	960	465	465	930	2840	20600	19883
5	5	15	2280	450	450	900	4100	23990	24787
10	5	15	4030	400	400	800	5650	27340	23596

§From Hammond *et al.*[1]

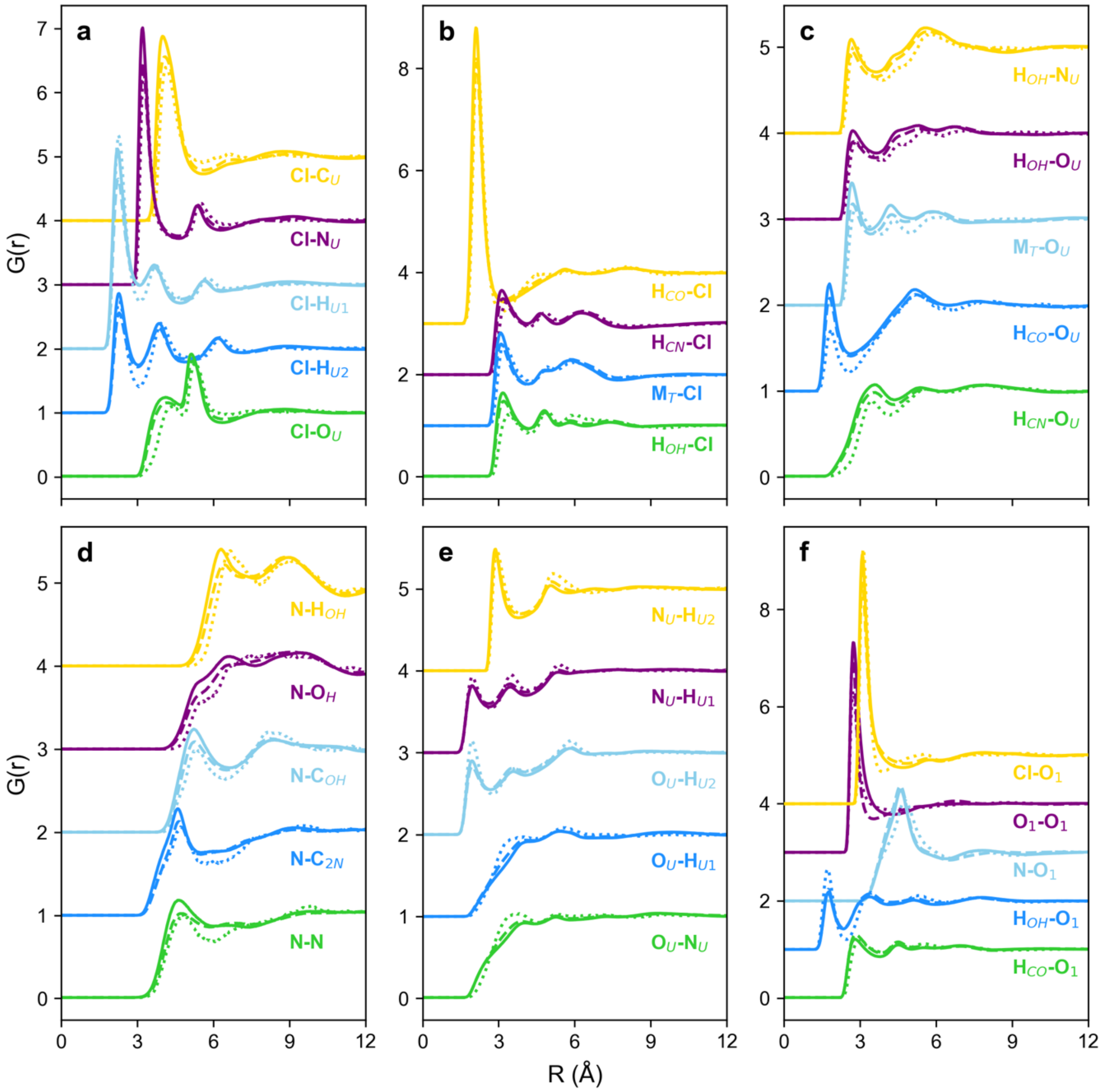

Fig. S1. Calculated partial radial distribution functions (pRDFs), for interactions targeted on chloride-urea (a), choline-chloride (b), choline-urea (c), choline-choline (d), urea-urea (e), and water (f). pRDFs are shown for ChCl:urea samples prepared with water contents of 2w (solid lines), 5w (dashed lines) and 10w (dotted lines).

Table S2. Complete set of calculated site-site partial coordination numbers (N_{coord}), iterated over the duration of the EPSR simulation. Literature data are shown for comparison, for systems without any dissolved cerium salt.

**‘A’**	**‘B’**	**R_{max} (Å)**			***N_{coord}***					
		2*w*	5*w*	10*w*	2*w*§	2*w*+Ce	5*w*§	5*w*+Ce	10*w*§	10*w*+Ce
Cl	C_U	5.0	5.1	5.2	2.93±1.40	2.94±1.25	2.40±1.35	2.40±1.26	1.79±1.24	1.84±1.22
Cl	N_U	3.9	4.0	4.1	2.77±1.59	2.81±1.49	2.28±1.53	2.27±1.46	1.71±1.39	1.75±1.39
Cl	H_{U1}	3.0	3.0	3.0	1.38±1.32	1.41±1.29	1.08±1.17	1.06±1.15	0.76±1.00	0.77±1.01
Cl	H_{U2}	3.0	3.0	3.0	0.89±0.86	0.90±0.87	0.65±0.77	0.65±0.76	0.44±0.65	0.45±0.65
H_{OH}	Cl	3.2	3.2	3.2	0.53±0.51	0.48±0.51	0.43±0.50	0.37±0.49	0.35±0.48	0.27±0.45
M_T	Cl	4.0	4.0	4.0	0.54±0.61	0.53±0.61	0.42±0.55	0.41±0.56	0.30±0.49	0.29±0.48
H_{CN}	Cl	4.0	4.0	4.0	0.55±0.60	0.51±0.60	0.44±0.56	0.39±0.54	0.33±0.51	0.28±0.48
H_{CO}	Cl	4.0	4.0	4.0	0.54±0.61	0.52±0.61	0.43±0.56	0.40±0.55	0.32±0.51	0.28±0.48
H_{CN}	O_U	3.5	3.5	3.5	0.45±0.61	0.50±0.62	0.33±0.54	0.36±0.55	0.22±0.45	0.22±0.45
M_T	O_U	3.4	3.4	3.5	0.48±0.63	0.54±0.65	0.36±0.55	0.40±0.57	0.25±0.47	0.26±0.48
H_{CO}	O_U	3.6	3.6	3.6	0.50±0.64	0.56±0.66	0.37±0.56	0.41±0.59	0.27±0.49	0.25±0.48
H_{OH}	O_U	2.5	2.5	2.5	0.17±0.38	0.18±0.39	0.13±0.35	0.13±0.34	0.10±0.30	0.09±0.29
H_{OH}	N_U	4.3	4.3	4.3	2.34±1.55	2.40±1.42	1.78±1.42	1.82±1.35	1.21±1.19	1.22±1.19
N	N	6.8	6.8	7.3	2.27±1.23	1.75±1.08	1.76±1.16	1.19±0.96	1.88±1.25	1.35±0.99
N	C_{2N}	7.5	7.5	7.7	4.53±1.50	4.02±1.35	3.85±1.49	3.24±1.24	3.41±1.43	2.85±1.14
N	C_{OH}	6.2	6.2	6.4	2.66±1.16	2.52±1.09	2.32±1.11	2.09±0.95	2.09±1.01	1.82±0.83
N	O_H	5.2	5.3	5.4	1.96±0.92	1.91±0.89	1.81±0.88	1.69±0.78	1.62±0.77	1.49±0.67
N	H_{OH}	5.6	5.6	5.8	2.29±1.06	2.22±1.02	2.01±0.98	1.87±0.87	1.84±0.90	1.67±0.78
O_U	N_U	3.5	3.5	3.6	3.19±1.01	2.98±0.90	2.96±0.96	2.82±0.87	2.78±0.89	2.73±0.86
O_U	H_{U1}	2.6	2.6	2.6	0.42±0.70	0.34±0.64	0.33±0.64	0.29±0.59	0.25±0.55	0.23±0.53
O_U	H_{U2}	2.6	2.6	2.6	1.72±0.85	1.61±0.83	1.58±0.83	1.57±0.79	1.51±0.77	1.51±0.75
N_U	H_{U1}	4.6	4.6	4.6	4.58±1.92	4.10±1.71	4.13±1.83	3.78±1.66	3.49±1.60	3.39±1.58
N_U	H_{U2}	4.6	4.6	4.6	4.69±1.71	4.23±1.50	4.19±1.65	3.88±1.48	3.57±1.45	3.44±1.41
Cl	Cl	6.0	6.0	6.0	1.14±0.90	1.32±1.26	0.85±0.80	1.05±1.16	0.58±0.69	0.76±1.03
N	O_1	5.6	5.4	5.4	3.65±1.90	3.82±1.89	6.82±2.41	7.46±2.16	10.12±2.42	11.31±2.19
H_{OH}	O_1	2.3	2.3	2.3	0.14±0.35	0.13±0.34	0.25±0.44	0.29±0.46	0.35±0.48	0.42±0.50
H_{CN}	O_1	3.5	3.6	3.6	0.53±0.70	0.54±0.70	1.13±0.95	1.31±0.95	1.61±1.05	1.93±1.03
H_{CO}	O_1	3.6	3.6	3.6	0.62±0.77	0.65±0.78	1.23±1.01	1.46±1.01	1.75±1.12	2.11±1.10
N_U	O_1	4.0	3.9	3.9	-*	1.24±1.06	-*	2.14±1.20	-*	3.36±1.31
C_U	O_1	5.2	5.2	5.2	-*	3.05±1.69	-*	6.10±2.00	-*	9.49±2.05
H_{U1}	O_1	2.4	2.4	2.4	-*	0.17±0.38	-*	0.31±0.48	-*	0.44±0.53
H_{U2}	O_1	2.4	2.4	2.4	-*	0.17±0.38	-*	0.32±0.49	-*	0.44±0.53
Cl	O_1	4.3	4.3	4.2	1.99±1.36	2.03±1.35	3.83±1.71	3.94±1.52	5.12±1.66	5.26±1.47
O_1	O_1	4.0	3.5	3.4	1.65±1.24	1.58±1.19	2.07±1.16	1.81±1.04	2.70±1.16	2.42±1.03

§From Hammond *et al.*[2]

*Urea-water site-site coordination numbers were not reported previously.[2]

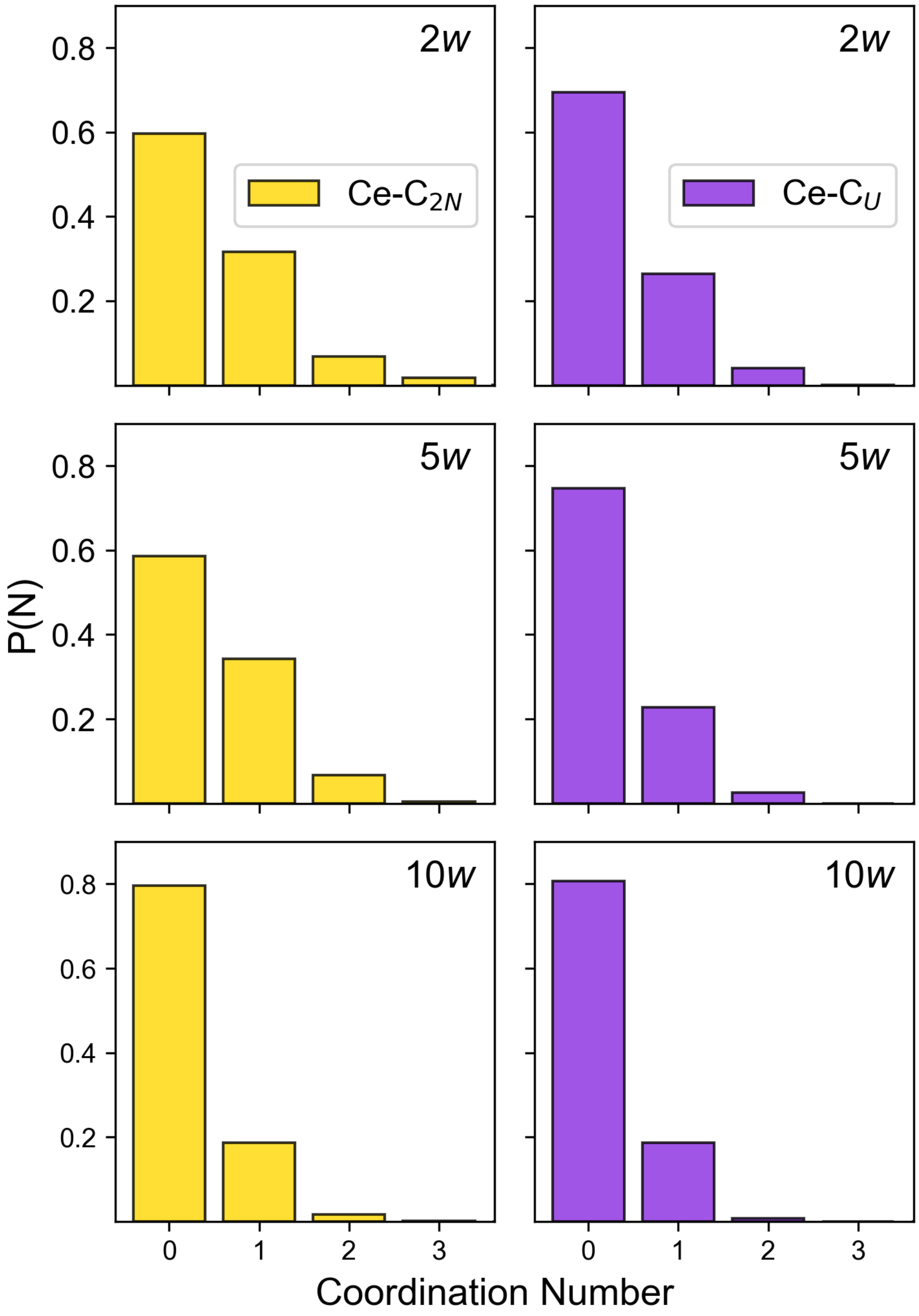

Fig. S2. Calculated coordination number histograms showing the probability of Ce^{3+} being coordinated by choline cations (yellow; left column) or urea HBDs (purple; right column), for the 2*w* DES (top), 5*w* DES (middle) and 10*w* DES (bottom).

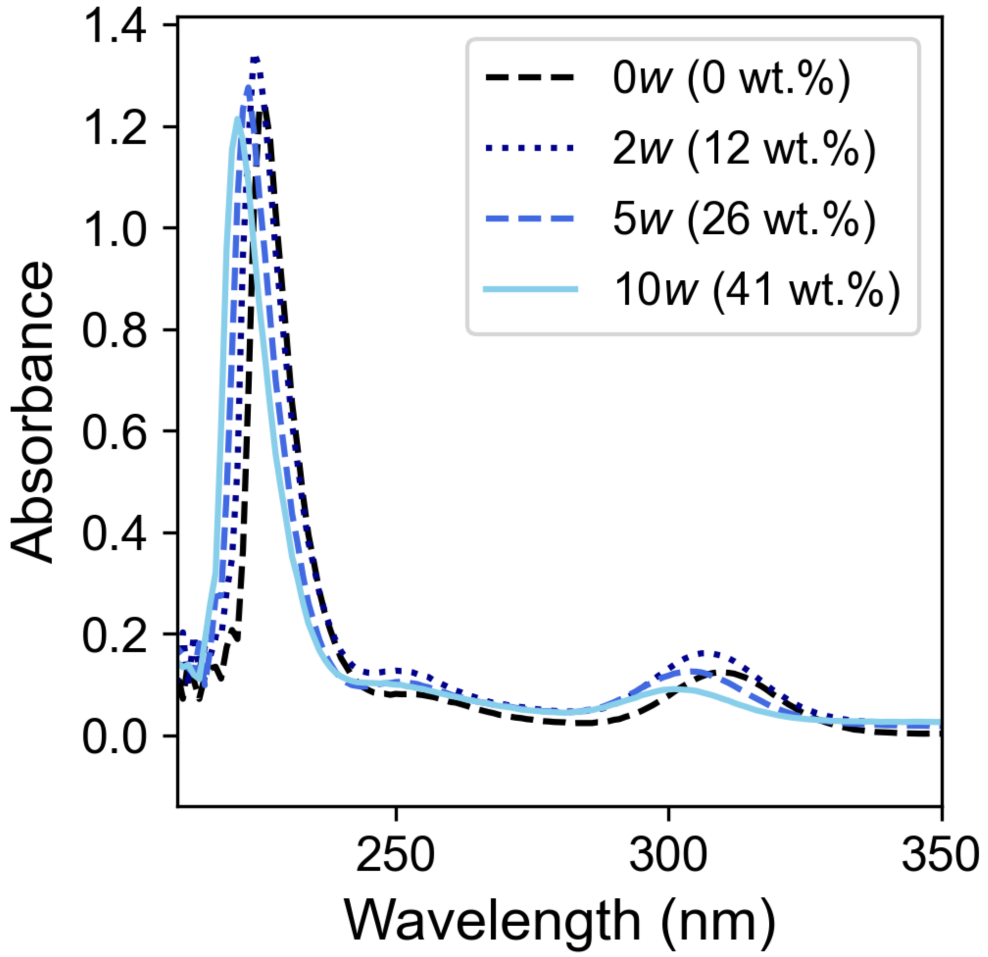

Fig. S3. UV-Vis spectroscopy measurements of dilute solutions of $Ce(NO_3)_3•6H_2O$ in the various DES-water solutions.

Table S3. Observed shift in UV-Vis absorbance peak maxima (±1 nm) as a function of water content in the prepared dilute $Ce(NO_3)_3•6H_2O$ DES-water solutions.

w	**Proposed complex**	***λ*₁ (nm)**	***λ*₂ (nm)**
0§	$[Ce(Cl)_{3.9}(Choline)_{1.1}(NO_3)_{0.5}(Urea)_{1.7}]^{3-}$ §	226	310
2	$[CeCl_6(H_2O)]^{3-}$	224	307
5	$[CeCl_5(H_2O)_2]^{2-}$	223	304
10	$[CeCl_5(H_2O)_3]^{2-}$	221	301

§From Hammond *et al*;[1] note that the proposed complex structure in the prior report is not directly comparable because the average structure was given, rather than the most probable coordination.

References

(1) Hammond, O. S.; Edler, K. J.; Bowron, D. T.; Torrente-Murciano, L. Deep Eutectic-Solvothermal Synthesis of Nanostructured Ceria. *Nat. Commun.* **2017**, *8*, 14150. https://doi.org/10.1038/NCOMMS14150.

(2) Hammond, O. S.; Bowron, D. T.; Edler, K. J. The Effect of Water upon Deep Eutectic Solvent Nanostructure: An Unusual Transition from Ionic Mixture to Aqueous Solution. *Angew. Chem. - Int. Ed.* **2017**, No. 56, 9782–9785. https://doi.org/10.1002/anie.201702486.
